# Performance assessment of the county healthcare systems in Kenya: a mixed-methods analysis

**DOI:** 10.1136/bmjgh-2020-004707

**Published:** 2021-06-24

**Authors:** Mark W Moses, Julius Korir, Wu Zeng, Anita Musiega, Joyce Oyasi, Ruoyan Lu, Jane Chuma, Laura Di Giorgio

**Affiliations:** 1 Independent Researcher; 2 School of Economics, Kenyatta University, Nairobi, Kenya; 3 Department of International Health, School of Nursing & Health Studies, Georgetown University, Washington, DC, USA; 4 Health Economics Research Unit, KEMRI-Wellcome Trust Research Programme, Nairobi, Kenya; 5 School of Public Health, Fujian Medical University, Fujian, China; 6 World Bank, Nairobi, Kenya; 7 World Bank, Washington, DC, USA

**Keywords:** health economics, health policy, health systems evaluation

## Abstract

**Introduction:**

A well performing public healthcare system is necessary for Kenya to continue progress towards universal health coverage (UHC). Identifying actionable measures to improve the performance of the public healthcare system is critical to progress towards UHC. We aimed to measure and compare the performance of Kenya’s public healthcare system at the county level and explore remediable drivers of poor healthcare system performance.

**Methods:**

Using administrative data from fiscal year 2014/2015 through fiscal year 2017/2018, we measured the technical efficiency of 47 county-level public healthcare systems in Kenya using stochastic frontier analysis. We then regressed the technical efficiency measure against a set of explanatory variables to examine drivers of efficiency. Additionally, in selected counties, we analysed surveys and focus group discussions to qualitatively understand factors affecting performance.

**Results:**

The median technical efficiency of county public healthcare systems was 84% in fiscal year 2017/2018 (with an IQR of 79% to 90%). Across the four fiscal years of data, 27 out of the 47 Kenyan counties had a declining technical efficiency score. Our regression analysis indicated that impediments to the flow of funding—measured by the budget absorption rate which is the ratio between funds spent and funds released—were significantly related to poor healthcare system performance. Our analysis of interviews and surveys yielded a similar conclusion as nearly 50% of respondents indicated issues stemming from poor budget absorption were significant drivers of poor healthcare system performance.

**Conclusion:**

Public healthcare systems at the county-level in Kenya general performed well; however, addressing delays in the flow of funding is a concrete step to improve healthcare system performance. As Kenya—and other countries—provides additional funding to meet their UHC goals, establishing a strong and robust public financial management system is critical to ensure that the benefits of UHC are realised.

**Key questions**

**What is already known?**

- Like many countries, Kenya continuously strives to improve access to quality and affordable healthcare; however, there exists very little evidence on actionable measures to improve the performance of the Kenyan public healthcare system.

**What are the new findings?**

- County public healthcare systems in Kenya performed relatively well as nearly three quarters of county-level public healthcare systems had a technical efficiency score greater than 80%.
- The impact of public financial management systems on the performance of healthcare systems has not been studied extensively in the literature; yet, in our analysis—which draws on both quantitative and qualitative research—we find that bottlenecks in the the flow of funding—proxied by the budget absorption rate—was a significant determinant of the performance of county public healthcare systems in Kenya.

**What do the new findings imply?**

- As Kenya—and countries around the world—develops policies and appropriate funding to scale up services to meet their universal health coverage goals, they must not overlook the need and important role that a strong and robust public financial management system plays.
- The analysis is an example of the complementary role both qualitative and quantitative research can play in answering important policy-relevant questions and finding actionable solutions.

## Introduction

Universal health coverage (UHC) aims to ensure that all individuals can obtain the healthcare they need without enduring financial hardship and the healthcare systems they seek care in are well-managed, adequately financed, and responsive to the needs of patients—even during healthcare crises like the COVID-19 pandemic.^1–5^ For more than a decade, the global health community mustered a concerted push towards achieving UHC. As countries pushed towards UHC, many countries experienced challenges in the rollout of their UHC policies.^4–12^ In this regard, Kenya is no different. One of Kenya’s first UHC initiatives was the establishment of the National Hospital Insurance Fund in 1967, which aimed to protect Kenyans from high healthcare costs,^13–15^ but in its initial formulation, it was beleaguered by weak governance structures, offered only a narrow benefits scheme and facilities and providers reported frequent delays in reimbursement.^13^ Despite new reforms, more reforms are likely necessary.^14 15^ More recently, Kenya has continually struggled to balance the need to generate revenue at healthcare facilities through the imposition of user fees with the need to reduce barriers to care; despite Kenya’s efforts, far too many Kenyans still face catastrophic levels of out-of-pocket healthcare spending and far too many healthcare facilities are in need of investment to adequately staff and stock clinics with needed medication and supplies.^14 16–18^ Kenya’s push towards UHC has not been futile: over the past 20 years, Kenya has made dramatic progress in reducing childhood deaths, improving access to maternal care and antiretroviral therapies and expanding health insurance coverage—especially to those most in need;^19 20^ nevertheless, UHC remains a distant goal in Kenya.^21^

In more recent years, the push towards UHC in Kenya gained momentum as the Kenyan president made UHC a central objective of his administration through his pledge to make quality healthcare services available to all Kenyans by the year 2022.^22 23^ Starting in 2018, the president’s UHC pledge began with the removal of all user fees at public healthcare facilities in four Kenyan counties.^22 23^ While his swift actions were lauded by many, including the Director-General of the WHO, critics argued the healthcare systems was unprepared for the sudden influx of patients seeking care at public healthcare clinics—leading to reported delays in care and shortages of drugs.^24^ These problems were compounded by ongoing healthcare worker strikes that demanded back pay and promotions and ultimately led to growing anxiety and low morale among healthcare workers.^25–28^

Kenya is a heterogeneous country—geographically, culturally and economically. The healthcare needs of each region within Kenya vary too as there are differential geographic patterns to diseases like malaria,^29^ diarrhea^30^ and undernutrition,^31^ and access to healthcare varies considerably with in-facility deliveries ranging from 33% to 100% between counties.^19^ To respond to the unique challenges within each county, Kenya devolved its healthcare system in 2013^32^ by transferring management of the healthcare system from the national government to each of the 47 county-level governments, except for national and teaching referral hospitals. In effect, this policy created 47 county-level public healthcare systems. Under this policy, both counties and the national government finance the public healthcare system but counties are charged with the responsibility of managing the healthcare system and delivering healthcare services. In a devolved healthcare setting like Kenya, public financial management systems play an even more important role as multiple private and government entities must interact and coordinate with one another to collect fees, issue reimbursements for care provided and distribute funds in a timely manner. While there has been increasing recognition of the role played by public financial management systems in the performance of the healthcare systems, this issue has not been examined in depth or in the context of efficiency analyses.

To make further progress towards UHC, there is a compelling need to improve the performance of the healthcare systems and make more efficient use out of the limited resources—especially since external support is expected to decline over the coming years.^20^ Recent evidence suggests the Kenyan healthcare system performs poorly as anywhere between 20% and 50% of the resources devoted to health in Kenya are used inefficiently.^33–37^ Critically, these studies focus primarily on measuring the performance of the healthcare system but provide little evidence on potential solutions to improve healthcare system performance. Looking across countries, solutions do exist to improve the performance of healthcare systems. These solutions include addressing inappropriate staff mix at healthcare facilities; overuse, underuse or unavailability of medicines and service offerings; poor quality of care and overall poor governance.^38^ In the present analysis, we evaluate healthcare system performance and potential remedies by taking a mixed-methods approach. We first quantitatively benchmark each of the 47 Kenyan county public healthcare systems against one another, then we analysed focus group discussions (FGD), interviews and survey responses to look for potential remediable drivers of poor healthcare system performance and then test these drivers within a quantitative framework.

## Methods

### Overview

Our analysis consisted of three steps. First, we estimated county healthcare systems’ technical efficiency—a measure that quantifies counties’ public healthcare systems ability to translate healthcare system resources into healthcare services and henceforth refers to this measure as healthcare system performance. We measured healthcare system performance by applying stochastic frontier analysis (SFA) to a panel dataset covering the fiscal years (FY) of 2014/2015 through FY2017/2018. As a second step, in selected counties with varying levels of measured healthcare system performance (high, medium and low), we surveyed healthcare system administrators and providers, held FGD and key informant interviews (KIIs) to understand, from their perspective, the drivers of poor healthcare system performance. Finally, we regressed our measure of healthcare system performance against a set of county-level covariates to explore the drivers associated with greater healthcare system performance. All analyses were conducted at the county level which served as the unit of analysis. Data used in the study were sourced from the District Health Information System 2, IntraHealth, the 2018 Kenyan Service Delivery Indicator survey and the 2018 Kenyan Household Health Expenditure and Utilization survey. The online supplemental appendix provides greater details regarding the data used in the analysis.

10.1136/bmjgh-2020-004707.supp1 Supplementary data

### Health systems’ efficiency estimates

We quantitatively estimated counties’ health system performance by employing a multioutput SFA model.^39–41^ SFA is a regression-based model used to measure how well county public healthcare systems provide healthcare services (eg, outpatient services and inpatient services) given the available set of inputs (eg, healthcare workers and healthcare resources). In our SFA models, we considered a range of important healthcare system outputs including total outpatient visits, total inpatient bed days and diagnostic and imaging services. The healthcare system inputs we considered included total beds and cots, total healthcare providers (eg, medical officers, clinical officers, and nurses), support care staff (eg, pharmacy staff and diagnostic and imaging staff), other staff (eg, administrators, hospital maintenance staff) and spending on drugs and value of drugs donated. We compared a variety of model specifications that combined inputs and outputs in a range of functional forms and evaluated these functional forms based on the plausibility of coefficients, information criterion tests and other measures of fit. This process led us to selecting a parsimonious Cobb-Douglas specification with two outputs (outpatient visits per capita, bed days per capita) and four inputs (healthcare providers, beds and cots, drug spending and value of drug spending per capita). All comparisons of SFA specifications may be found in the online supplemental appendix.

### Determinants of efficiency analysis

We analysed the factors associated with county healthcare performance by regressing the logit transformed measure of technical efficiency against a set of covariates capturing factors thought to be associated with healthcare system efficiency in a within-between model.^42^ The within-between model, also sometimes referred to as a hybrid model, allows for the estimation of both between effects—time-invariant covariates that explain contextual differences between counties—and within effects—time-varying covariates that are robust to omitted or unobserved time or county invariant factors. Asymptotically, the within coefficients of the within-between model are equivalent to the coefficients of a traditional within model, commonly referred to as a fixed effect model.

The within-between model suited our investigation well as it allowed us to control for time-varying covariates as well as a set of potentially useful explanatory covariates with only 1 year of observation. If we assumed that the covariates with only a single year of observation were representative across the time period of analysis, we could include these covariates as time-invariant covariates in the between portion of our within-between model. The inclusion of these time-invariant covariates allowed us to investigate covariates’ contextual relationships with healthcare system performance across counties—an investigation that would not be possible in a within model as time-invariant covariates would be absorbed by county-level effects.

Our data on healthcare services provided were often incomplete as mean reporting rate across all counties was 87%. Incomplete reporting may bias our measure of healthcare system performance as not all healthcare services provided by a healthcare system would be captured and thus reducing its measured healthcare system performance—all else being equal. We adjusted for incomplete reporting by including log reporting rate as a covariate in our within-between model exploring drivers of poor healthcare system performance. Using the within coefficient of facility reporting rate, we created county-year specific adjustment factors using the following formula:

$$Reporting\;Rate\;Adjustment\;Factor_{i,t}=\beta *\left(log\left(100\%\right)-log\left(r_{i,t}\right)\right)$$

where β is the coefficient on the natural log of health facility reporting and $r_{i,t}$ is the county-year observed reporting rate. Because we logit transformed our measure of technical efficiency to bound predictions from our regression between zero and one, we recovered technical efficiency scores adjusted for incomplete reporting using the following formula:

$$TE_{i,t}^{\prime }=logit\;(Reporting\;Rate\;Adjustment\;Factor_{i,t}+logit\;(TE_{i,t}){)}^{-1}$$

where $TE_{i,t}$ was the county-year specific measure of technical efficiency and $TE_{i,t}^{\prime }$ was the county-year specific measure of technical efficiency adjusted for incomplete reporting.

In our investigation of the determinants of efficiency, we found a robust and consistent signal which indicated that county-level HIV/AIDS prevalence and public health facility usage were significant contextual determinants of county healthcare system performance. While county-level HIV/AIDS prevalence is and should be a responsibility of the public healthcare system in the long run, in the short run, a high prevalence of HIV/AIDS in the county population likely results in more difficult and time-intensive healthcare encounters which require more resources on the part of the county public healthcare system. Further, public healthcare facility usage is within the control of counties’ public healthcare systems, but variation in usage across counties is likely due in large part to uncontrollable factors such as viability and consequently availability of private healthcare facilities within counties and ability of county residents to afford care provided at private healthcare facilities.

To address these contextual issues that were outside the immediate control of healthcare systems, we standardised all county technical efficiency scores based on HIV/AIDS prevalence and public facility usage. This standardisation process mirrored our adjustment for incomplete reporting—the only difference was that our adjustment for HIV/AIDS prevalence and public healthcare system usage was based on the mean of each variable across counties, opposed to a 100% reporting rate used to adjust for incomplete reporting. Unless otherwise noted, our reported estimates of technical efficiency adjust for incomplete reporting, HIV/AIDS prevalence and public facility usage.

### Focus group discussions, key informant interviews, and survey analysis

In five counties, we conducted FGD, KIIs and administered surveys to healthcare providers and administrators. These five counties were selected using initial result from our SFA analysis. Two of the counties performed relatively well on our measurement of healthcare system performance, two counties performed relatively poorly and one county performed near the median. In each county, we invited members of the county health management team, staff at hospitals, health centres and dispensaries (eg, administrators, clinical officers, providers and nurses) to participate in FGDs. Additionally, we selected members of county health management teams and key staff (providers and other administrators) to participate in KIIs. Topics covered during FGDs and KIIs included governance, accountability, leadership, financial management, access to care and health system delivery. Recordings from these discussions were transcribed and reviewed. We invited all participants in FGD and KIIs to participate in a survey. The administered surveys asked respondents to assess, on a numeric scale from zero to six, how beneficial resolving specific healthcare system issues would be in improving healthcare system efficiency. To garner honest and truthful responses, participants’ identities and titles were kept anonymous. In total, we held 8 FGDs that ranged in size from 7 to 10 people, 58 KIIs and surveyed 104 individuals. A detailed analysis of these data may be found elsewhere.^43^

## Results

### Data

In table 1, we present FY 2017/2018 county-level data across a selection of indicators. Across counties, Makueni had the highest outpatient public healthcare utilisation rate at 2.19 annual outpatient visits per capita and Kisumu had the highest inpatient bed-day utilisation rate at 0.30 annual bed days per capita. Lamu had the highest density of healthcare providers at 1.54 providers per 1000 and Isiolo had the highest density of beds at 1.83 per 1000. The urban county of Nairobi had the lowest outpatient utilisation rate at 0.52 outpatient visits per capita; Laikipia and the northern county of Mandera had lowest inpatient bed-day utilisation rate at 0.001 bed days per capita. In addition to having the lowest inpatient bed-day utilisation rate, Mandera had the least available public healthcare resources with 0.30 healthcare providers per 1000 persons and 0.05 beds per 1000 persons.

**Table 1:** County-level and national summary statistics from fiscal year 2017/2018

County	Outpatient visits per capita	Bed days per capita	Healthcare providers per 1000 persons	Beds per 1000 persons	Adjusted technical efficiency (%)	Annual percentage change in adjusted technical efficiency (%)
Baringo	1.08	0.03	0.88	0.50	78	−1.56
Bomet	1.46	0.02	0.66	0.13	95	−0.20
Bungoma	0.86	0.09	0.59	0.78	79	−5.02
Busia	1.16	0.08	0.77	0.68	91	−0.54
Elgeyo Marakwet	1.96	0.03	1.04	1.22	89	0.53
Embu	1.86	0.02	1.23	0.41	90	−0.81
Garissa	1.51	0.11	0.63	0.65	93	3.97
Homa Bay	1.07	0.04	0.92	0.82	81	−2.90
Isiolo	1.35	0.10	1.36	1.83	75	5.77
Kajiado	0.96	0.02	0.58	0.36	83	2.81
Kakamega	1.32	0.08	0.75	1.04	87	−1.19
Kericho	1.23	0.08	0.83	0.48	92	0.34
Kiambu	1.33	0.08	0.94	1.03	84	−2.25
Kilifi	0.77	0.09	0.57	0.67	79	−0.21
Kirinyaga	1.72	0.09	0.88	1.45	87	−0.61
Kisii	0.88	0.05	0.69	0.56	78	−4.94
Kisumu	1.26	0.30	0.98	1.42	89	0.19
Kitui	1.86	0.05	1.38	0.94	87	4.88
Kwale	1.54	0.05	0.66	0.94	93	−1.26
Laikipia	1.30	0.01	1.21	0.16	65	2.17
Lamu	1.60	0.1	1.54	1.19	81	1.00
Machakos	1.38	0.03	0.85	0.77	88	−1.23
Makueni	2.19	0.07	1.44	1.00	90	1.67
Mandera	0.90	0.01	0.30	0.05	83	18.34
Marsabit	1.55	0.03	0.81	0.37	92	5.20
Meru	0.78	0.08	1.41	0.60	75	−2.20
Migori	1.15	0.04	0.79	0.75	84	−3.22
Mombasa	0.68	0.08	0.82	0.98	60	1.04
Muranga	1.25	0.02	0.75	0.75	90	−1.48
Nairobi	0.52	0.07	0.45	0.34	81	0.38
Nakuru	1.23	0.11	0.71	1.38	82	−1.30
Nandi	1.17	0.02	0.63	0.39	83	−1.99
Narok	0.56	0.03	0.42	0.39	68	−3.97
Nyamira	1.08	0.04	1.04	0.59	67	−8.26
Nyandarua	1.20	0.04	0.66	0.45	90	−0.09
Nyeri	1.93	0.19	1.32	1.32	93	0.61
Samburu	1.04	0.01	0.91	0.45	61	−8.72
Siaya	1.58	0.02	0.81	0.51	92	0.70
Taita Taveta	1.53	0.08	1.37	1.00	85	−2.10
Tana River	1.09	0.01	0.83	0.08	90	10.59
Tharaka Nithi	1.54	0.04	1.47	1.06	86	−3.07
Trans Nzoia	0.57	0.08	0.57	0.43	69	−6.36
Turkana	0.86	0.07	0.51	0.35	87	0.27
Uasin Gishu	1.52	0.14	0.64	1.31	93	0.61
Vihiga	1.04	0.02	0.59	0.90	69	−4.47
Wajir	1.61	0.02	0.65	0.37	83	14.74
West Pokot	1.02	0.04	0.65	0.41	84	0.76
Kenyan population weighted average	1.14	0.07	0.79	0.72	83	−0.49

Estimates of adjusted technical efficiency accounting for HIV/AIDS prevalence, public healthcare facility utilisation and incomplete reporting rate. Unadjusted technical efficiency estimates as well as technical efficiency estimates that are only adjusted for reporting rate may be found in the online supplemental appendix.

### Efficiency results

Furthermore, in table 1, we report our estimates of technical efficiency—our measure of healthcare system performance. In FY 2017–2018, the median county technical efficiency was 84% with an IQR between 79% and 90% (table 1). Bomet had the highest measured technical efficiency at 95% in 2017–2018, but nine counties (about 1 in five counties) also had technical efficiency scores greater than 90% (table 1). Overall, Mandera had the highest annual percentage growth in measured technical efficiency at 18.34% per year, but 57% of counties (27 out of 47 counties) had declining technical efficiency scores over the 4-year study period. Our estimates of technical efficiency that adjusted for reporting rate, HIV/AIDS prevalence and public health facility usage had a 0.91 correlation with estimates of technical efficiency that only adjusted for reporting rate.

**Figure 1:**
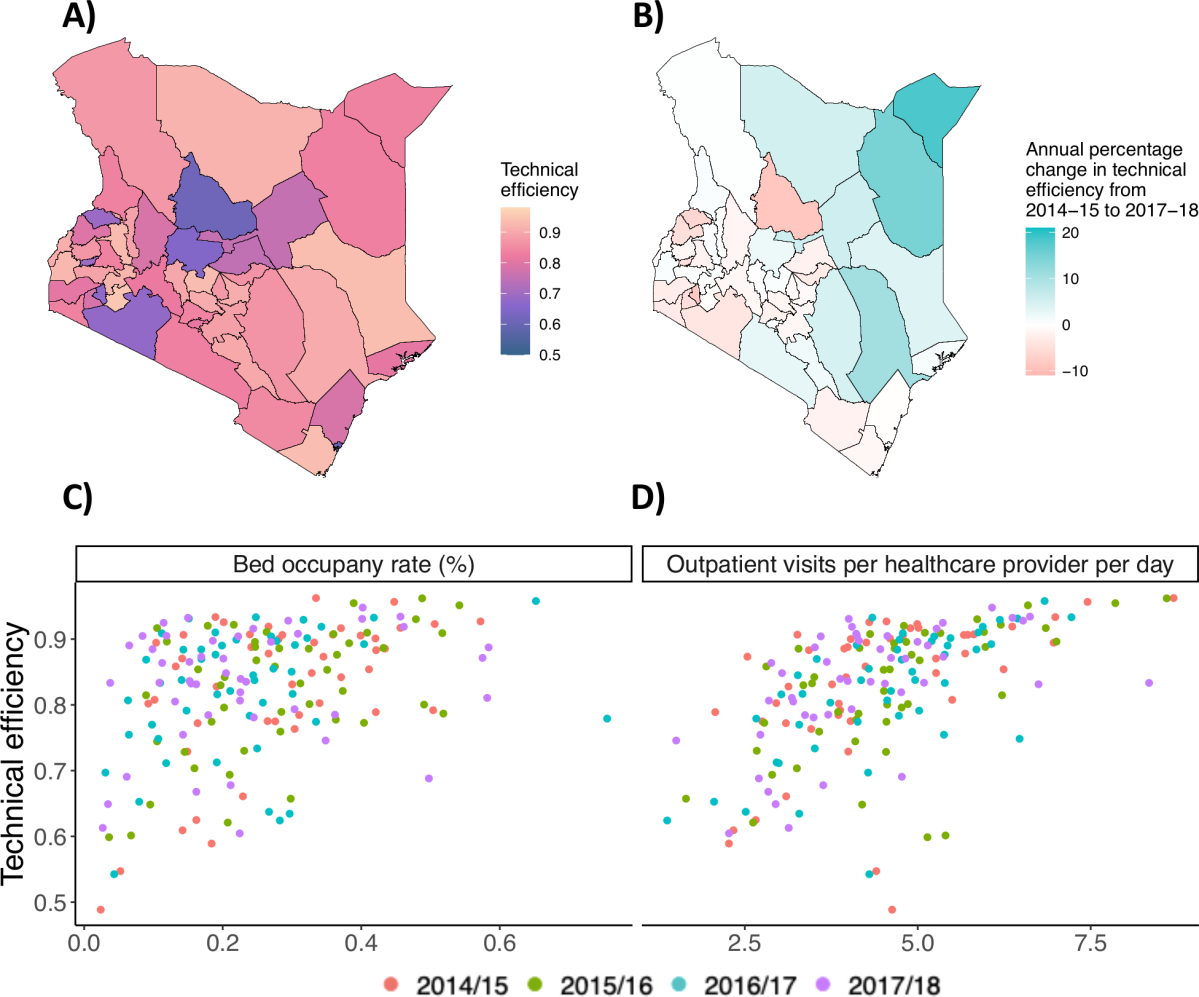
Map of county-level technical efficiency, rate of change in technical efficiency and comparisons of technical efficiency to other common measures of healthcare system performance. Panel A displays results from fiscal year 2017/2018. Panels B, C and D display results from fiscal year 2014/2015 to fiscal year 2017/2018. Note that panels C and D compare adjusted technical efficiency to patient volume data where incomplete reporting is likely prevalent.

We mapped our FY 2017/2018 measurement of technical efficiency in figure 1A. The best performing counties were geographically scattered from the west (Bomet) to the north east (Garissa) and to the south east (Kwale), while the urban county of Mombasa and central counties (Lapika and Samburu) had the lowest efficiency scores. Northern counties like Wajir and Mandera had the fastest improvement in healthcare system performance during the study period (figure 1B).

In figure 1C, D, we scattered traditional measures of healthcare system performance and capacity (bed occupancy rate and outpatient caseload) against our adjusted measure of technical efficiency. County bed occupancy rates showed very little correlation with healthcare system performance (Pearson correlation *r*=0.20); however, there existed a high level of correlation between outpatient caseload and our measure of healthcare system performance (Pearson correlation *r*=0.66).

### Determinants of efficiency

Results from various model specifications of our within-between regression model analysing the determinants of efficiency may be found in table 2. In our first specification, we tested a wide range of covariates which included the ratio of doctors to all other healthcare providers, the prevalence of poverty, self-rated health, availability of drugs and donor involvement, measured as the ratio of the value of donated drugs to total drug spending (inclusive of donated drugs and drugs purchased with domestic funds). We removed these covariates due to lack of statistical significance and created a slightly more parsimonious model in the second specification and showed factors like absenteeism and provider diagnostic accuracy, and stunting prevalence had no statistically significant association with healthcare system performance either. In our third specification, we found the ratio of outpatient visits to inpatient bed days, a measure approximating case-mix intensity, was not statistically significant at the 95% CI.

**Table 2:** Regression specifications for determinants of efficiency analysis

Predictors	-1		-2		-3		-4	
	Estimates	P value	Estimates	P value	Estimates	P value	Estimates	P value
*Within (time-varying)*								
log reporting rate	1.20	**0.005**	1.24	**0.003**	1.21	**0.003**	1.25	**0.002**
log budget absorption rate	0.41	**0.003**	0.45	**0.003**	0.39	**0.004**	0.38	**0.006**
log total spending on health per cap	0.25	0.300	0.23	0.351				
log ratio of outpatient visits to inpatient bed days	−0.12	0.080	−0.13	0.062	−0.13	0.066		
log ratio of value of donated drugs to overall drug spending	0.05	0.461						
log ratio of doctors and clinical officers to other healthcare staff	−0.34	0.580						
*Between (time-invariant)*								
Mean of log reporting rate	2.53	0.077	2.28	**0.040**	2.68	**0.009**	2.51	**0.008**
Mean of log budget execution rate	−0.27	0.612	−0.41	0.350	−0.43	0.236	−0.41	0.251
Mean of log total spending on health per cap	−0.42	0.381	−0.28	0.453				
Mean of log ratio of outpatient visits to inpatient bed days	0.18	0.231	0.20	0.146	0.16	0.207		
Mean log ratio of value of donated drugs to overall drug spending	−0.01	0.681						
Mean of log ratio of doctors and clinical officers to other healthcare staff	0.51	0.539						
log out-of-pocket spending per consultation at public facility	−0.69	**0.023**	−0.70	**0.001**	−0.59	**<0.001**	−0.60	**<0.001**
log HIV/AIDS prevalence	−0.21	0.273	−0.18	0.263	−0.24	**0.043**	−0.25	**0.028**
log public healthcare facility utilisation	1.38	0.212	1.75	**0.031**	1.32	**0.048**	1.41	**0.019**
log access to healthcare facility	0.06	0.681						
log fraction of total facilities that are primary care facilities	3.47	0.442						
log poverty rate	−0.10	0.812						
log of self-reported health	−0.20	0.453						
log diagnostic accuracy	0.79	0.434	0.80	0.339				
log absenteeism	0.42	0.534	0.55	0.309				
log stunting prevalence	−0.52	0.265	−0.29	0.457				
log medical equipment availability	−0.20	0.561						
log pharmaceutical availability	0.22	0.818						
R2 conditional / R2 marginal	0.781/0.378		0.771/0.375		0.764/0.374		0.766/0.377	
AIC	302.615		292.7		285.309		278.694	

Bolded values indicate p-values less than or equal to the p-value of 0.05.

Additional specifications may be found in the online supplemental appendix. Within covariates were also specified as between covariates by taking their mean across the panel.

AIC, Akaike information criterion.

In our preferred model specification, the fourth specification in table 2, the within coefficients indicated higher facility reporting rates and improved budget absorptions rates—measured by the ratio between funds spent and the funds released—were positively associated with healthcare system performance at greater than the 95% confidence level. In addition, the between coefficients in our model—which attempt to explain associations across counties opposed to associations within counties—showed that out-of-pocket spending per consultation and HIV/AIDS prevalence were significant and negatively associated with healthcare system efficiency while public healthcare facility utilisation was positively associated with health system performance and significant at the 95% confidence level. Notably, the marginal R^2^—which measures variation explained by fixed effects—never reached over 40%, while the conditional R^2^—which measures variation explained by fixed effects and county-level effects—explained over 75% of variations. This conclusion implies that there are still many unknown county-level drivers of poor healthcare system performance. Additional model specifications may be found in the online supplemental appendix.

### Key informant interviews and focused group discussions

Table 3 presents select quotes from discussions with interviewees and participants in FGD. Participants in our interviews and discussions primarily noted that the lack of available funding curtailed the healthcare facilities ability to operate effectively. Lack of funding prevented healthcare facilities from conducting outreach activities and led to delays in procurement of commodities and eventual stockouts. Interviewees noted that the availability of medical commodities was essential to providing high quality care—and when facilities experienced stockouts, their patient volumes fell. A stockout at one healthcare facility due to lack of funds led one facility manager to illegally divert funds to continue the operations of the facility. In other facilities, stockouts degraded the morale of staff—many of whom were already overworked and were not motivated due to the absence of promotional opportunities.

**Table 3:** Selected quotes from interviewed healthcare providers and administrators

Topic	Quote
Physical access	*‘other clients who are near here but during the time of rain, there is a river, that over flows, it makes accessibility a problem because there is no bridge’*
Budget execution	*‘Because we usually rely on the national treasury to release the funds to the counties. So, you find that most of the time [national treasury] delays in dispersing the funds, so sometimes we get a delay in procuring our health commodities. In such instances we get shortages but somehow, we try to curb by using the Facility Improvement Fund (FIF) for the bigger facilities’†*
	*‘(I would like] A system that does not require me to break the law and sign for cash that I’m not supposed to sign. I’d prefer a system where I do not have to be there for it to run, you see. I’d prefer a system where allocations like Linda mama (insurance program to fund maternal care) go to what Linda mama is supposed to do. Not diverting funds to go assist and manage the hospital’†*
Lack of funds	*‘we encourage people to get NHIF (National Hospital Insurance Fund) because that means the treatment will be free if they are cover is up to date so I feel like we have made that effort. And currently we collect a lot of, actually most of the money we use to run the hospital comes from the NHIF claims. Yeah because we…we …we haven’t received money from the county, in almost three years now’†*
	*‘Yes to some extent in terms of the projects that we are implementing. Sometimes officers need to be on the field and you cannot go to the field when you do not have access to maybe the funds or the facilitation that you require’*
Stock outs	*‘(when] we don’t have supplies, we don’t have drugs you find that our patients actually go down, the patients number goes down. Once we get the supplies all the hospital will be filled up’*
	*‘The delays in payment. We procure but then they stay even three months before paying the suppliers and the supplier says we can’t supply you until our debt is cleared’*
	*‘to improve on the services, we actually need more supplies because the supplies that we receive from KEMSA (Kenya Medical Supplies Authority), sometimes they normally bring us a fixed budget, they do not bring what we ordered maybe because of the financial constraints from the county’†*
Motivation	*‘(Regarding stock outs] it is demoralizing because patients have confidence in you and you have nothing to offer, you are losing the confidence you have’*
	*‘We have never gone without pay. At least the relation is okay. At least every monthly we get our salaries.’†*
	*‘Maintaining of personnel, you know staff need to be motivated. Like work with promotions, if promotions are done in time, they will really motivate staff…You know the board is an independent body, and when they do their promotions they don’t tell us why they have promoted these ones, why they have not promoted the others’†*
Staffing levels	*‘like the number of staff, of nurses is really not really good it really needs a lot to be done so we have a shortage of nurses. So, if nurses are increased it will really help in burn out because current cadre of nurses is really beyond the required standard it would improve the performance’*
	*‘If we do not have enough budget, you cannot employ [staff), and start the process of determining how much budget is needed for health in terms of human resources’†*
Absenteeism	*‘Those [absenteeisms] are very rare cases, very rare cases and even then we do have an advisory committee that deals with all the disciplinary issues and they are very effective though we don’t get such not unless they are very adamant (Laughs)’*

Excerpts were from transcribed interviews of healthcare providers and administrators in five Kenyan counties. All quotes from key informant interviewees are denoted with a †; all other quotes were from focus group discussions.

### Survey results

**Figure 2:**
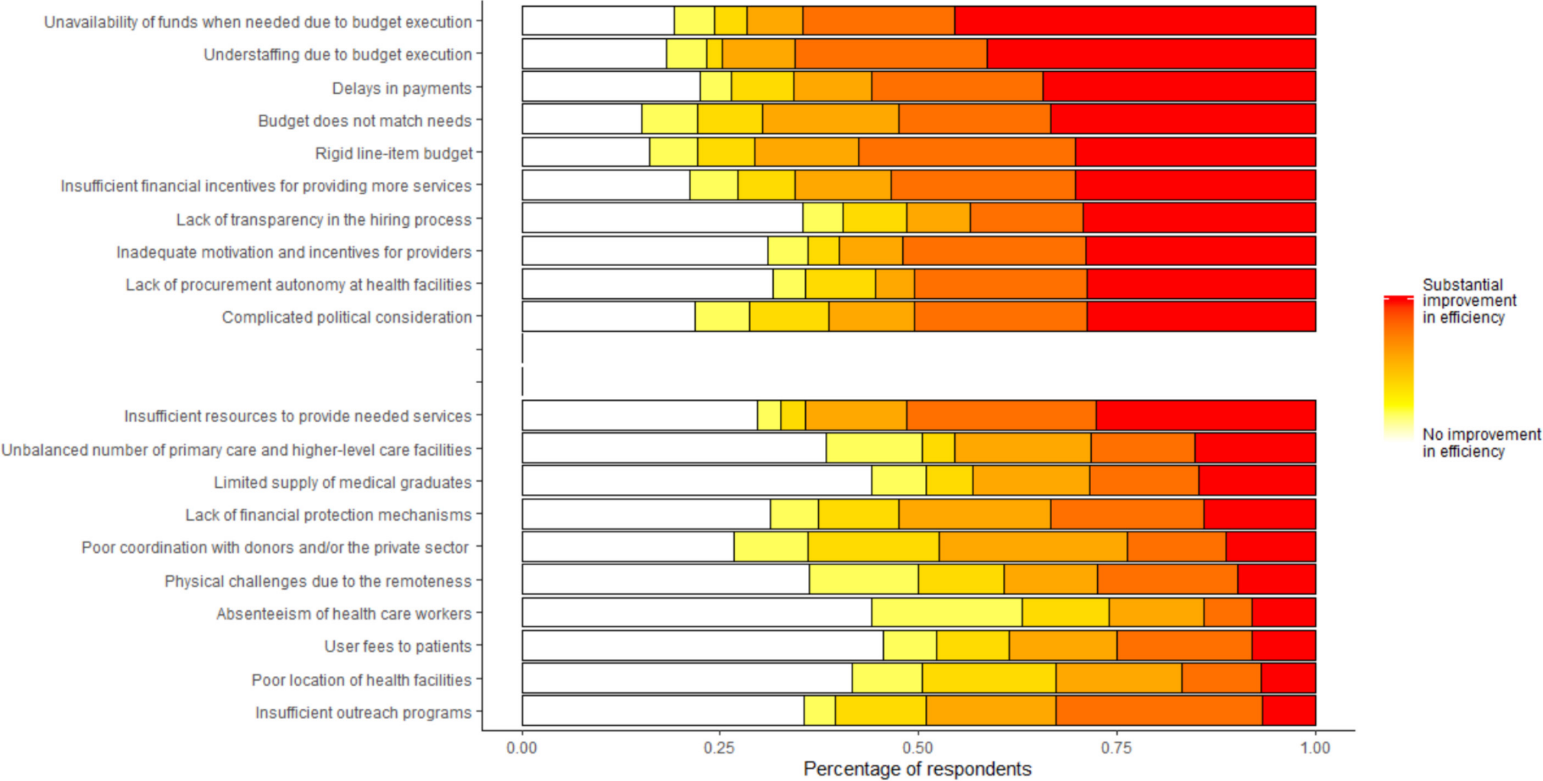
Survey responses from healthcare providers and administrators. Healthcare providers and administrators in five Kenyan counties were surveyed and asked to rate each factor on a scale of one to six. Panel A displays the top 10 factors respondents said most contributed to poor health healthcare system performance. Panel B displays a selection of other factors commonly cited as contributors to poor healthcare system performance.

Figure 2A summarises survey results from those that participated in interviews and FGDs. We display the top 10 factors survey respondents said could most substantially improve the performance and efficiency of their county’s public healthcare system. The top three factors respondents felt could most substantially improve the performance all related to the flow of funding: 35% of respondents indicated that improving the timeliness of available funds would substantial improve performance of healthcare systems; nearly 45% of respondents felt that addressing the lack of available funds due to poor budget execution could lead to substantial improvements in healthcare system performance and over 40% respondents believed that public healthcare systems could substantially improve their performance by addressing understaffing issues that arose due to poor budget execution.

In addition to the flow of funding, respondents reported that they had little control of the funds they received which impacted their ability to provide care. Over 30% of respondents believed that the rigid line-item budgets and the mismatch of the budgetary items with healthcare needs of their counties resulted in poor health system performance. Further, another 30% of respondents felt the lack of transparency in hiring processes and inadequate financial incentives to motivate providers to deliver more healthcare services led to poor health system performance.

In figure 2B, we present a selection of factors commonly associated with poor health system performance that were outside the top 10 factors respondents considered most responsible for poor health system performance. Nearly 50% of respondents believed that addressing issues surrounding patient user fees and provider absenteeism would not lead to substantial improvements in the performance of healthcare systems. Potential issues related to remoteness of healthcare facilities, poor economic conditions of surrounding community or lack of outreach activities were largely not viewed as drivers impacting healthcare system performance nor were issues related to coordination between external and private providers, fragmentation of providers, management of resources at healthcare facilities or uncertainty related to funding from external resources. Survey responses were generally in agreement across all five counties as the measured correlation coefficient across counties exceeded 0.60.

## Discussion

In FY 2017–2018, we estimated 72% of all county-level public healthcare systems in Kenya had a technical efficiency score greater than 80%—and 26% of counties had a technical efficiency score greater than 90%. While these results are encouraging, the results do highlight select county public healthcare systems have significant room for improvement. Our determinants of efficiency analysis indicated bottlenecks in the flow of funding—proxied by the budget absorption rate—significantly impaired healthcare system performance, a conclusion supported by survey responses and interviews with healthcare providers and administrators.^43^ Nevertheless, our determinants of efficiency regression model only explained 37% of all variation, suggesting the existence of many other unknown factors associated with poor healthcare system performance.

A well-performing county-level public healthcare systems in Kenya is vital to ensuring that every Kenyan has access to essential healthcare services and the limited resources devoted to healthcare are used efficiently. As the Kenyan healthcare system tackles the COVID-19 pandemic and continues to pursue UHC initiatives, now more than ever it is important to evaluate healthcare systems and discover potential solutions to address poor healthcare system performance.

Despite severe budgetary pressure from the COVID-19 pandemic, in FY2020/2021 the Kenyan government committed over 12.6 billion Ksh (US$126 million) to fund the rollout of UHC.^44 45^ While publicly committing to investing in healthcare systems and approving budgets that reflect these commitments is a necessary step towards UHC, the effect of these investments may be muted if the funds do not quickly flow through the necessary financial channels and result in actual health expenditure. According to our data, the national budget absorption rate—measured as the ratio between the funds spent and the funds released—in Kenya for FY 2018/2019 was 94%. Although this rate is higher than other countries,^46 47^ it does not reflect the full extent of the problem as the figure summarises annual expenditure—and not delays in expenditures occurring within a fiscal year.

Bottlenecks in the flow of funding, like poor budget absorption, is often due to rigid or slow procurement processes, poor communication between relevant entities, complex reimbursements, continual budget revisions or generally poor financial organisation and planning.^47 48^ Our determinants of efficiency analysis indicated that bottlenecks in the flow of funding related to poor budget absorption was significantly associated with impaired healthcare system performance. This conclusion was supported by survey responses which indicated that healthcare providers and administrators most often believed issues stemming from bottlenecks in the flow of funding like delays in payment or lack of available funds was the major source of healthcare system inefficiency. Interviewees noted the lack of available funds delayed implementation of healthcare services, created difficulties with procuring necessary drugs and supplies and the hiring of new staff leading to significant understaffing issues. When faced with limited funds, a healthcare facility manager said they had to make the decision to divert funds from other revenue streams to finance facility operations. Managers prioritised using the limited available funds to pay staff salaries and relied on suppliers’ goodwill to extend facilities credit for drugs and necessary supplies. This procurement process was unsustainable because, as debt accrued, suppliers became increasingly unwilling to offer healthcare facilities drugs and supplies on credit—leading to eventual stock outs.

Prior work suggested bottlenecks in the flow of funding—and more broadly poor financial management—contributed to poor healthcare system performance in other countries like Mexico, Nepal, Ghana, Nigeria and Tanzania.^49–53^ In Mexico, an evaluation of Seguro Popular—a social insurance programme for those most in need—cited the decentralisation of the Mexican healthcare system as a cause of funding bottlenecks. Similar to Kenya, a portion of the Mexican healthcare system was decentralised in an effort to increase the autonomy of local healthcare authorities, allowing them to tailor their services to meet the unique needs of their communities. But a byproduct of decentralisation was the creation of additional layers of bureaucracy that impeded the flow of funds. Further, the additional layers of bureaucracy made tracking the flow of funding more challenging, decreasing the transparency and accountability of healthcare system financing. To the best of our knowledge, our study is the first analysis to offer statistical evidence linking bottlenecks in the flow of funding to poor healthcare system performance.

Interviewees and survey respondents noted rigid line-item budgets combined with delays in receiving funds limited the ability of facility managers to purchase necessary supplies to provide care, resulting in patients seeking care elsewhere. We found no significant association between healthcare system performance and drug and equipment availability. The lack of a significant association may be due to analysing healthcare performance at the county level—opposed to the healthcare facility level. While individual healthcare facilities’ performance may be dependent on drug and equipment availability, the signal at the county level may be diminished as patients simply seek care at facilities adequately stocked with drugs and equipment elsewhere in the county. Qualitative evidence suggested that patient volumes fluctuated with availability of medicines and equipment as one interviewee noted, ‘[when] we don’t have drugs you find that our patients actually go down…Once we get the supplies all the hospital will be filled up’. Stockouts affect patients’ health and provider morale as well as one provider noted stockouts were, ‘demoralizing because patients have confidence in you and you have nothing to offer, you are losing the confidence you have’.

In recent years, the public healthcare system in Kenya faced several extended healthcare worker strikes that cited understaffing as their chief complaint.^54^ Interviewees noted that a shortage in funding prevented the hiring of new staff. Continued understaffing leads to burnout, especially among nurses. This conclusion is supported by survey respondents—over 40% of whom felt that understaffing of the healthcare system substantially impacted healthcare system performance. Unfortunately, we had no quantitative measure of understaffing and thus were unable to test this observation within our determinants of efficiency analysis.

Relatedly, absenteeism is a factor commonly cited in the literature as a contributor to poor healthcare system performance.^55 56^ Yet, when we regressed absenteeism against our measure of healthcare system performance, we found no statistically significant result (p value consistently greater than >0.30). The lack of a significant finding may suggest that our covariate of absenteeism lacks construct validity: our covariate measuring absenteeism indicated 27 out of the 47 Kenyan countries had an absenteeism rate of 50% or greater^18^ while interviewees and surveyed healthcare providers and administrators indicated absenteeism was uncommon.

In addition to absenteeism, nearly 50% of surveyed healthcare workers and administrators believed that user fees assessed to patients were not a contributor of poor healthcare system performance. Despite our survey respondents’ perceptions, our analysis indicated higher out-of-pocket payments per consultation at public facilities was associated with lower healthcare system performance. This result is likely reflective of reduced barriers to care that lead to greater healthcare utilisation and consequently a higher score on our measure of healthcare system performance base on patient volumes.

A concern of ours was that differential levels in quality of care provided by the healthcare systems would confound our estimates of healthcare system performance. Providing higher quality of care requires additional resources that could otherwise have been dedicated to providing more healthcare services with poorer quality of care. Accounting for quality of care provided could reduce our estimates of healthcare system performance, compared to not controlling for quality of care. Despite this, controlling for quality of care is uncommon in benchmarking studies. To the best of our knowledge, only two SFA studies of healthcare system performance in Sub-Saharan Africa attempted to control for quality of care. The studies found that controlling for quality of care had little to no impact on the overall measurement of technical efficiency.^36 57^

We explored the relationship between quality of care and healthcare system performance using potential proxies of quality of care like providers’ diagnostic accuracy, availability of drugs or equipment; however, these proxies were shown to have no significant relationship with healthcare system performance. The ratio of healthcare providers trained as doctors to all other healthcare providers—potentially suggestive of quality of care provided or intensity of care—had no significant association with healthcare system performance either. With the available quality of care proxies, we found no quantitative evidence suggesting quality of care confounded our measure of healthcare system performance.

Kenya is also one of the largest recipients of development assistance for health in the world—over 20% of healthcare spending in Kenya is financed by development assistance.^58^ These funds purchase needed medication for HIV/AIDS, malaria and tuberculosis and they provide direct financial support to hire staff and fund the adoption of health information systems. The benefit of these additional resources may spill over into the broader healthcare system and serve to improve healthcare system performance. Alternatively, if these additional resources do not lead to a proportional increase in patient utilisation, then a county may perform relatively poorly on our measure of healthcare system performance as these additional resources are not being used to their full potential, all things being equal. In our determinants of efficiency analysis, we proxied donor involvement at the county level by the fraction of drugs financed by donors over the value of all drugs used in each county. We found no substantive quantitative signal relating donor involvement with our measure of healthcare system performance.

A chief concern of ours was under-reporting of service volumes (outpatient visits or inpatient bed-days) in our data. While we accounted for under-reporting using facility reporting rates in our determinants of efficiency analysis, this adjustment likely did not capture all of the bias in the data. Future work must go into validating the completeness of the data we draw on as well as the utility of the data for research purposes. Moreover, continued efforts must be made to gather data on potential, remediable drivers of healthcare system performance. The regression we implemented in our determinants of efficiency analysis explained less than 40% of all observed variation—suggesting the potential existence of unknown, influential drivers of healthcare system performance. Our work identified poor budget absorption as a culprit of poor healthcare system performance but delays in expenditure and disbursement of funds can compound due to delays occurring elsewhere (eg, treasury, ministry of health or county-level). Our data did not allow us to explore potential sources of delays, but the exercise would be a valuable and worthwhile investigation in future studies.

## Conclusion

Overall, our analysis indicated that Kenyan healthcare systems performed relatively well, but a select number of counties could still improve on their performance. Well-functioning public healthcare systems are central to making progress towards UHC and addressing the COVID-19 pandemic. As the Kenyan healthcare system—and healthcare systems around the world—tackles these two challenges, establishing a well-functioning financial management system to timely and entirely disburse funds is critical to facilitating progress towards achieving UHC. Overlooking or taking for granted the financial management system can deleteriously impair healthcare systems and their ability to protect individuals’ health.

## Data Availability

Estimates and related data are available upon request. However, it should be noted that a portion of the data data used in the study were obtained from a third party or through personal communication, and we do not have the necessary permission to make these data publicly avaialble. In circumstance where we do not have the permission to release the data, we can provide the contact information to parties with the authority to grant access.
