# Supplementary material for: Performance assessment of the county healthcare systems in Kenya: a mixed-methods analysis
Source: BMJ Glob Health. 2021 Jun 24;6(6):e004707. doi: 10.1136/bmjgh-2020-004707 (PMC8230973; doi:10.1136/bmjgh-2020-004707)
Supplement: Supplementary data [file bmjgh-2020-004707supp001.pdf]

Appendix to *Assessment of Kenya's county healthcare systems: a mixed-methods analysis*

Data

In table #1 below we display the sources of relevant data used in the analysis. It is worth noting that IntraHealth provided data on staffing levels for the most recent fiscal year (FY) but no historical data on staffing levels existed, only historical attritions and new hires. We calculated historical staffing levels (e.g. FY 2016/17 staffing levels) by subtracting new hires and adding attritions to FY 2017/18 staffing levels; we repeated this process to calculate FY 2015/2014 and FY 2015/16 staffing levels. Extreme outliers (e.g. order of magnitude difference from adjacent years) accounted for less than 1 percent of total data. These data were replaced with an imputed value that was calculated with county means and time trends. Additionally, we collected data on county-level expenditure that were sourced from county government expenditure reports, and data on measures like HIV/AIDS prevalence, stunting, poverty rate, public facility usage, and access to health facility were sourced from the 2018 Kenyan Household Health Expenditure and Utilization Survey, while measures such as absenteeism and diagnostic accuracy were sourced from the 2018 Kenyan Service Delivery Indicator survey.

Table #1

Data	Source
Service volumes (outpatient, inpatient bed days, inpatient admissions, laboratory test performed, imaging performed) and number of beds and cots	DHIS2
Staffing levels (Number of clinical officers, nurses, other medical care staff, administrators, and non-medical staff)	IntraHealth
Drugs purchased and donated	Kenya Medical supplies authority
Health financing and approved budget data	Country and Government Expenditure Reports
diagnostic accuracy, absenteeism, medical equipment availability, pharmaceutical availability	Kenya Service Delivery Indicator Survey, 2018
Out-of-pocket spending, HIV/AIDS prevalence, public healthcare facility utilization, poverty rate, self-reported health, stunting prevalence	Kenya Household Health Expenditure Utilization Survey, 2017-18

Methods

As mentioned in the main text, when analyzing the specifications of the stochastic frontier analysis (SFA) model, we took into consideration the plausibility of the coefficients in the SFA model, their significance, and Akiake information criterion (AIC). In Table #2 we present estimated coefficients of a sampling of model specifications we considered. Note that when computing standard errors, we clustered on counties.

Table 2

	(1)		(2)		(3)		(4)		(5)	
Covariates	*Estimates*	*p*	*Estimates*	*p*	*Estimates*	*p*	*Estimates*	*p*	*Estimat*	*p*
log ratio of bed days to outpatient visits	-0.138	0.026	-0.138	0.028	-0.138	0.030	-0.138	0.006	-0.545	<0.001
log beds per capita	0.204	0.046	0.206	0.084	0.203	0.076	0.204	0.015	1.189	0.002
log medical care staff per capita	0.316	0.004	0.315	0.004	0.360	0.015	0.001	0.001	-4.170	<0.001
log purchased drugs per capita	0.469	0.043	0.046	0.053	0.455	0.045			-0.172	0.002
log donated drugs per capita	0.008	0.798	0.007	0.817	0.156	0.653			-0.720	0.015
log ratio of imaging services to diagnostic services					-0.001	0.964				
log support staff per capita					-0.045	0.674				
log total drug spending per capita (purchased and donated)							0.035	0.288		
log ratio of bed days to outpatient visits squared									-0.540	<0.001
log beds per capita squared									0.065	0.006
log medical care staff per capita squared									-0.308	<0.001
log purchased drugs per capita squared									0.035	<0.001
log donated drugs per capita squared									0.062	<0.001
Year Dummies										
2015	0.014	0.597	0.013	0.614	0.003	0.926	0.004	0.874	-0.053	0.057
2016	-0.050	0.188	-0.050	0.191	-0.071	0.246	-0.071	0.074	-0.161	0.002
2017	-0.137	0.004	-0.136	0.004	-0.162	0.041	-0.150	0.002	-0.018	0.000
Parameters of one sided distribution										
mu			-0.148	0.797						
sigma u	-2.275	<0.001	-2.054	0.004	-2.270	<0.001	-2.520	<0.001	-2.762	<0.001
sigma v	-4.063	<0.001	-3.985	<0.001	-4.060	<0.001	-3.670	<0.001	-23.990	<0.001
Distribution of inefficiency	half-normal		truncated normal		half-normal		half-normal		half-normal	
AIC	-1.597		0.267		1.98		2.51		-60.64	

We ultimately implemented specification 1. This decision was supported by the fact that it appeared the truncated normal distribution of inefficiency implemented in specification 2 had no real advantage compared to the half normal assumption of the distribution of inefficiency implemented in specification 1. When we included additional covariates such as imaging service or other staff aside from medical care staff (doctors, clinical officers, and nurses), these covariates had no impact as the covariates' p values were quite large. When we condensed spending on drugs into one covariate, the covariate lost significance and the AIC value deteriorated. In the fifth specification tested, we implemented a quadratic model. While this specification had a lower AIC value, the coefficient on important covariates such as medical care staff and drug spending had implausible values.

Determinant of efficiency

In Table 3 we present alternative specifications of our determinants of efficiency regression. Note, in the specifications presented below we added specific covariates of interest to our preferred model specification.

Table 3

	(1)		(2)		(3)		(4)		(5)	
Predictors	*Estimates*	*p*	*Estimates*	*p*	*Estimates*	*p*	*Estimates*	*p*	*Estimat*	*p*
Within (time-varying)										
log reporting rate	1.250	**0.002**	1.250	**0.002**	1.250	**0.002**	1.250	**0.002**	1.250	**0.002**
log budget execution rate	0.380	**0.006**	0.380	**0.006**	0.380	**0.006**	0.380	**0.006**	0.380	**0.006**
log total spending on health per cap										
log ratio of outpatient visits to inpatient bed days										
log ratio of doctors and clinical officers to other healthcare staff										
Between (time-invariant)										
Mean of log reporting rate	2.520	**0.008**	2.530	**0.008**	2.350	**0.016**	2.720	**0.005**	2.760	**0.010**
Mean of log budget execution rate	-0.390	0.280	-0.430	0.236	-0.370	0.313	-0.460	0.196	-0.390	0.276
Mean of log total spending on health per cap										
Mean of log ratio of outpatient visits to inpatient bed days										
Mean of log ratio of doctors and clinical officers to other healthcare staff										
log out-of-pocket spending per consultation at public facility	-0.540	**0.003**	-0.630	**0.001**	-0.610	**<0.001**	-0.590	**<0.001**	-0.620	**<0.001**
log HIV/AIDS prevalance	-0.250	**0.028**	-0.250	**0.036**	-0.220	0.070	-0.240	**0.039**	-0.270	**0.025**
log public healthcare facility utilization	1.350	**0.026**	1.480	**0.017**	1.530	**0.015**	1.240	**0.040**	1.310	**0.039**
log access to healthcare facility										
log fraction of total facilities that are primairy care facilities	2.450	0.415								
log poverty rate										
log of self-reported health										
log diagnostic accuracy			0.370	0.612						
log absenteeism					0.310	0.493				
log stunting prevalence										
log medical equipment availability							-0.300	0.188		
log pharamceutical availability									0.350	0.597
R^2 conditional / R^2 marginal	0.781 / 0.378		0.771 / 0.375		0.764 / 0.374		0.766 / 0.377		0.766 / 0.373	
AIC	302.615		292.7		285.309		278.694		279.44	

Alternative efficiency estimates

The Excel file accompanying this appendix contains three versions of estimates of technical efficiency for all counties and across all fiscal years. The three versions are (1) efficiency estimates without adjusting for reporting rate, public facility utilization, or HIV/AIDS prevalence; (2) technical efficiency estimates controlling for only reporting rate; (3) and technical efficiency estimates controlling for reporting rate, HIV/AIDS prevalence, and public facility utilization.
